# Advances and Challenges in Minimally Invasive Spine Surgery

**DOI:** 10.3390/jcm13113329

**Published:** 2024-06-05

**Authors:** Timothy Y. Wang, Michael Y. Wang

**Affiliations:** Department of Neurological Surgery, University of Miami Hospital, Miami, FL 33136, USA; tyw3duke@gmail.com

**Keywords:** minimally invasive, spine surgery, technology, advancements, challenges

## Abstract

Minimally invasive spine surgery continues to grow and develop. Over the past 50 years, there has been immense growth within this subspecialty of neurosurgery. A deep understanding of the historical context and future directions of this subspecialty is imperative to developing safe adoption and targeted innovation. This review aims to describe the advancements, and challenges that we face today in minimally invasive spine surgery.

## 1. Introduction

In the early days of spine surgery, even small operations such as lumbar discectomies required large incisions, extreme muscle dissection and tissue destruction. This was a result of several limitations, including lack of fundamental anatomic understanding, limited surgical instruments, lack of enabling technology, poor visualization, and primitive technique. As surgeons began recognizing the significant morbidity of such endeavors, there was a strong push towards minimizing collateral damage to tissues and other essential structures, while still accomplishing the same goals as traditional open surgery. This fueled the birth of modern minimally invasive spine surgery (MISS). Minimally invasive spine surgery has undergone immense growth within the last 50 years. Technological and software advancements, especially within the last 20 years, have ushered in a new era of ultra-minimally invasive surgery, including endoscopy and augmented reality. In this manuscript, the authors seek to provide a scoping review of minimally invasive surgery, with an emphasis on historical context, current technology, and future directions.

## 2. Developmental Milestones

**Figure 1:**
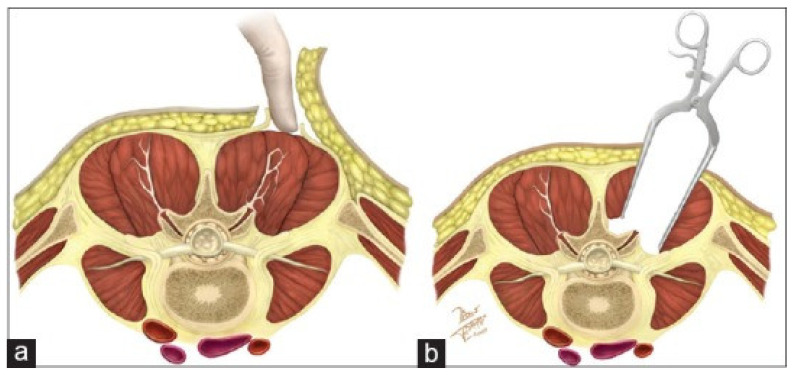
Adapted from Guiroy et al. 2018 [1]. (**a**) The intermuscular plane is developed between the medial multifidus and the lateral longissimus. (**b**) This exposes the facet and transverse process and lateral laminar surface. Permission obtained under the terms of the Creative Commons Attribution-NonCommercial-ShareAlike 3.0 License.

The late 1960s and early 1970s served as a crucial time for the development of minimally invasive principles. A pivotal orthopedic surgeon-innovator at that time, Leon L. Wiltse, recognized the need for a less traumatic avenue to the lumbar spine. His seminal 1968 paper describing the paraspinal sacrospinalis-splitting method is the first to describe access to the lumbar spine through intramuscular planes instead of through standard subperiosteal exposure. This exposure exploited the natural muscular plane between the multifidus, spinalis and longissimus muscles, and could access the facet joint and lamina and allow for decompression with less blood loss and muscle destruction than the standard midline incision (Figure 1 [1]). At the time of this manuscript, Dr. Wiltse’s paper has over 600 citations and to this day, the “Wiltse Approach” remains a fundamental corridor for disorders of the lumbar spine [2].

**Figure 2:**
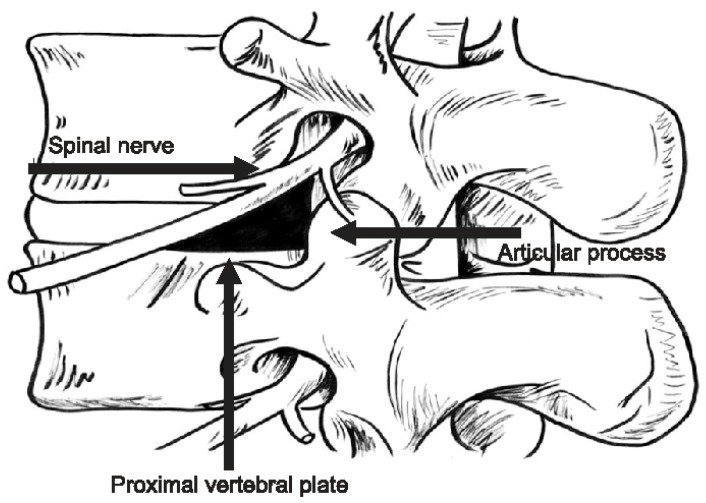
Adapted from Park et al. 2011 [3], the dimensions and borders of Kambin’s triangle are defined by the exiting nerve root as the hypotenuse, followed by the superior articular process medially and the superior endplate of the caudal vertebral body, inferiorly. Permission obtained under the terms of the Creative Commons Attribution-NonCommercial-ShareAlike 3.0 License.

In the 1980s, Parviz Kambin utilized a similar plane for percutaneous access to the lumbar disc space. In his technical report, he utilized a paramedian incision approximately 8 cm off midline to obliquely enter the disc space through a prism bound by the exiting nerve root laterally, superior endplate inferiorly, and superior articulating process medially (Figure 2 [3,4]). Dr. Kambin described this approach using fluoroscopy to aid disc access, which ushered in an era where fluoroscopy, as opposed to direct visualization, could be used for spinal pathologies. Using this approach, Dr. Kambin could then aspirate disc fragments through negative pressure. He would go on to publish his results in the first 100 patients, demonstrating an 87% success rate utilizing this new technique [5]. Today, Kambin’s triangle continues to be a workhorse approach for percutaneous access to the lumbar disc space, and can now also be used for interbody fusion [6].

Another pioneer surgeon of this era was Robert W. Williams. Williams, a private-practice surgeon based in Las Vegas (NV, USA), began experimenting with microsurgical techniques in the cervical and lumbar spine with the goal of accessing the lumbar spine and disc space through as small an incision as possible. His work, initially presented in the American Association of Neurological Surgeons (AANS) and Congress of Neurological Surgeons (CNS) meetings were initially met with caution and skepticism; however, his techniques slowly gained traction and interest, first within orthopedic spine surgery, and then subsequently with neurological spine surgeons. In his pivotal paper published in 1978, he discussed a microsurgical technique for the virgin lumbar herniated disc that featured no laminectomy or curettage of the disc space and focused on preservation of extradural fat and blunt perforation of the disc annulus. His successful experience with 530 patients over a 5.5 year follow-up is considered one of the first major technical papers within minimally invasive surgery [7].

Minimally invasive spine surgery continued to evolve over the ensuing decades. The turn of the new millennium brought explosive change and evolution to minimally invasive surgery. High-definition surgical microscopes, 3-dimensional CT-navigation and endoscopy brought about an entirely new subspecialty within spinal surgery, ushering in a new era of technical development.

Synonymous with minimally invasive surgery is tubular spine surgery. In this muscle sparing approach, either a fixed diameter or expandable tube is placed through a paramedian incision through the erector muscles and is docked on the lamina or laminar-facet junction. Tubular approaches were also particularly useful for far lateral disc herniations. Once the tube is docked and its position confirmed with fluoroscopy, the surgeon can then perform maneuvers with the high-speed drill, Kerrison rongeurs, and nerve root retractors as they would with traditional open surgery. One of the first series describing the utility of tubular systems was published in 1999 by Kevin Foley. In this report, Foley demonstrated the docking of the tubular system on the junction of the cephalad transverse process and the pars interarticularis. Decompression of the exiting nerve root could then be completed by shaving the superior articular process with a combination of high speed drills and Kerrison rongeurs [8]. Additional modifications and usage of endoscopic cameras also allows for adaptation to the cervical spine. In 2002, Richard Fessler and Larry Khoo described their experience in utilizing this technology for posterior cervical foraminotomies. In their series published in 2002, patients undergoing tubular posterior cervical foraminotomies had equivalent clinical outcomes compared to standard open approaches; however, tubular exposure resulted in less blood loss, shorter hospitalizations, and less pain medication usage [9]. Usage of tubular systems was then applied to a variety of lumbar pathologies and is now used to perform ipsilateral–contralateral decompressions [10], treat infections [11], remove tumors [12] and perform interbody fusions [13].

As tubular techniques gained popularity, there was a drive to better optimize visualization. This time-period saw new technological developments within fiber-optics, glass rod endoscopy, image processing, angled endoscopes, and endoscopic spinal instrumentation. The introduction of endoscopy to spinal surgery further reduced collateral tissue damage and ushered in a new era of ultra-minimally invasive spine surgery.

In 1983, Forst and Hausmann published their landmark paper on “nucleoscopy”, a technique utilizing a modified endoscope that fitted down the working channel of an existing tubular system. While this system focused mainly on visualization, this study is credited as one of the first to apply endoscopy to the field of spine surgery [14]. Schreiber and Suezawa utilized endoscopic discectomy instruments to then perform nucleotomies with continuous endoscopic visualization. In their 1989 case series on 109 patients, they demonstrated a 72.5% success rate, even when applied to patients with spondylolisthesis or revision disc herniations [15].

The development of endoscopically-compatible curettes, rasps, and drills further expanded the applications for endoscopic spine surgery. In their 2005 technical paper, Schubert and Hoogland described a novel foraminotomy technique through Kambin’s triangle. Utilizing reamers and curettes, they were able to expand the dimensions of Kambin’s triangle and decompress the exiting nerve root by removing the ventral portion of the superior articulating process [16]. This was further validated by Yeung and Tsou and Jasper, Francisco and Telfian, who each described small modifications to the endoscopic foraminotomy technique [17,18].

Isolated foraminal pathology, however, is relatively uncommon within the lumbar spine. Thus, while existing endoscopic techniques could provide patients with excellent foraminal decompression, there were few patients who were appropriate operative candidates. Endoscopic spine surgeons soon recognized the need for endoscopic decompression of the central canal. This spurred the development of the interlaminar endoscopic technique [19]. Through the interlaminar space, spine surgeons could remove the ligamentum flavum to provide central decompression, thus greatly expanding the utility of endoscopic spine surgery. Surgeons then found methods to perform partial facetectomies and access the disc space for discectomy and interbody placement, all through the interlaminar space and through an incision less than 1 cm long [20]. Additional advancements in endoscopic spine surgery include bi-portal endoscopy [21], posterior cervical endoscopic foraminotomy [22], anterior cervical endoscopic discectomy [23], and thoracic endoscopic discectomy [24], which have expanded the endoscopic literature profile from fewer than 50 publications in 2010 to more than 250 by 2020 [25].

**Figure 3:**
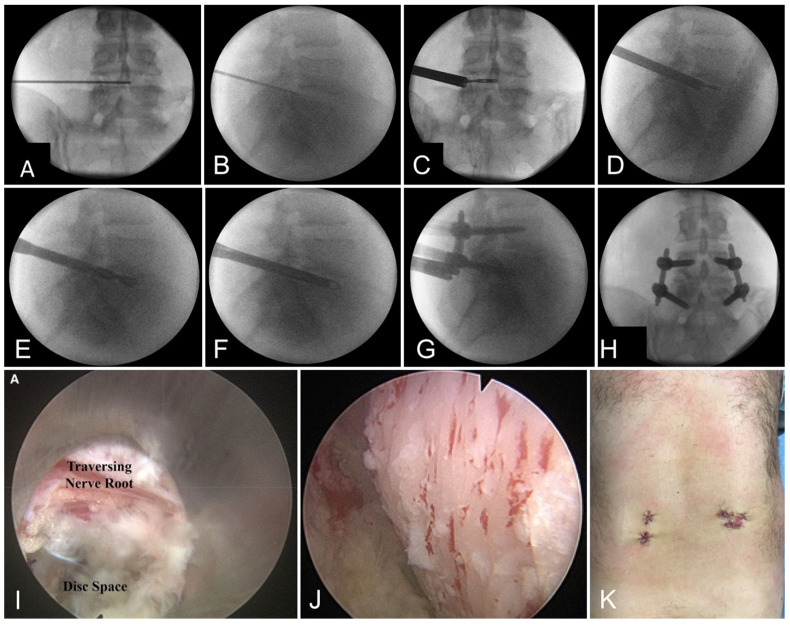
Fluoroscopy-guided awake endoscopic transforaminal lumbar interbody fusion. In Panel (**A**,**B**), the disc space is accessed through Kambin’s triangle using a spinal needle and nitinol wire. In Panel (**C**,**D**), the endoscopic portal is placed into the disc space after sequential dilation. In Panel (**E**,**F**), a combination of brushes and curettes are used to prepare the endplates, which can be visualized as seen in Panel (**I**,**J**). Placement of percutaneous screws, as well as an interbody implant, is performed using the surgeon’s preferred systems (in this case, Optimesh, Spineology, Panel (**G**,**H**)). The surgery is completed through a series of stab incisions, as seen in Panel (**K**). Panel (**I**,**J**) adapted from Wang et al. [28,29]. Remaining Figure adapted from Yoon et al. [29]. Published with permission.

The small incisions and reduced tissue destruction afforded by minimally invasive surgery spurred an additional wave of innovation, namely awake spine surgery. Awake spine surgery had already been routinely performed for orthopedic procedures (utilizing spinal anesthesia or regional blocks) and cranial surgery (i.e., awake tumor resections, deep brain stimulation). While the usage of spinal anesthesia in lumbar disc surgery was first described in 1959 [26], it was not until the1990s that spinal anesthesia became a more widely accepted practice [27]. The spinal surgeries carried out awake were limited at that time to laminectomies and discectomies, in part due to a lesser degree of stimulation and more consistently predictable operative times, the latter being important due to the limited duration of spinal anesthesia. However, as fusion surgery adopted percutaneous and endoscopic techniques, surgeons realized that lumbar fusion could be performed hyper-efficiently with minimal blood loss and minimal collateral tissue damage. In 2016, Wang and Grossman published the first ever series of lumbar fusions in patients sedated with local (non-spinal) anesthesia and a gentle infusion of propofol and ketamine. Their first ten patients treated had an average surgical time of 113 min with an average blood loss of 65 cc and an average length of stay of 1.4 nights (Figure 3 [28,29]). Importantly, no patients required conversion to general anesthesia. Since then, there have been a multitude of additional studies validating awake lumbar spine fusions [30,31].

As we enter the 21st century more deeply, the authors anticipate further development and refinement of minimally invasive techniques. The development of enabling technologies, such as robotics, augmented reality, and 3D preoperative nerve segmentation, will be paramount to the evolution of minimally invasive spine surgery (Table 1).

## 3. Challenges to Adoption

There is no definitive milestone that formally announced the arrival of minimally invasive spine surgery. However, most modern spine surgeons would be likely to point to the early 2000s as the “take-off” of minimally invasive techniques. Nowadays, it is estimated that up to 900,000 cases a year could be performed using existing minimally invasive approaches [32]. In the early 2000s, however, the novelty of minimally invasive spine surgery meant a significant learning curve and distrust of novel technologies. For example, a survey performed by Hartl et al. in 2007 reported that only 11% of surgeons were utilizing intraoperative navigation, with those using it citing its utility in performing complex cases, increasing surgeon accuracy, and reducing radiation exposure. At that time, intraoperative navigation had been used for more than ten years [33], but there was obviously distrust in its utility. Interestingly, those survey participants from countries with fewer medical resources cited lack of minimally invasive equipment, such as the availability of intraoperative navigation, as a major detriment to adoption of minimally invasive surgery [34]. Other cited barriers for widespread adoption included high up-front investment and increased radiation exposure [34].

Equally important to the growth of minimally invasive spine surgery was the availability of quality education, training, and mentorship. However, at the time, there were few surgeons who regularly applied minimally invasive surgery to their patient population, and many of these surgeons practiced in settings without trainees. As a result, there were few young surgeons who received the appropriate mentorship on the technical nuances, complications, and decision-making behind minimally invasive surgery [35]. As a result, early cases using minimally invasive techniques resulted in increased operative times and unforeseen complications. This tempered enthusiasm and required the surgeon to learn an entirely new set of troubleshooting algorithms for each new technique. As minimally invasive spine surgery has grown immensely since the early 2000s, surgeons looking to specialize in minimally invasive techniques must now master completely novel surgical approaches, including lateral lumbar interbody fusion [36], posterior tubular surgery [37], oblique lumbar interbody fusion [38], percutaneous fusion [39,40], and now even endoscopic approaches [41].

The evolution of the lateral lumbar interbody fusion is a prime example of this growth curve. Initially introduced in the early 2000s and then formally published in 2006, the lateral approach to the spine introduced an entirely new dimension of treatment for lumbar spondylosis and spinal deformity. After entering the retroperitoneal space, the surgeon expands a retractor through the psoas muscle allowing for a complete discectomy and placement of a large footprint interbody implant. When first introduced, many surgeons were hesitant to adopt this technique as this approach required unfamiliar patient positioning and involved an entirely new series of operative maneuvers. The lateral interbody fusion also relied heavily on indirect decompression through ligamentotaxis and foraminal expansion through disc height elevation, which at the time was an unproven concept [42]. Additionally, surgeons began experiencing previously unheard of or rare complications to spine surgery including ureter injury [43], bowel injury [44], pseudo-hernia [45], psoas hematoma [46], femoral nerve palsy [47], and aortic or inferior vena cava injuries [48,49]. These complications deterred many early adopters from continuing with this approach. After further refinements in technique and a focus on surgeon education and training, however, lateral lumbar interbody fusion is now considered an important asset in the spine surgeon’s armamentarium and can be used for significant sagittal and coronal deformity correction [50,51].

The rapid rise of novel technologies is often accompanied by financial incentives for surgeons, thus introducing new ethical concerns for these techniques. One such approach was the axial lumbar interbody fusion (AxLIF). This approach was described as a technique that addressed L5-S1 pathologies through a minimally invasive paracoccygeal approach, and initial studies reported decreased blood loss and faster operative times compared to open traditional techniques [52]. However, as more surgeons adopted this approach, reports of significant perioperative complications, such as rectal perforations and associated infections, began to surface [53,54,55]. Long term follow-up then began revealing high rates of pseudo-arthrosis and a high proportion of patients fused in the ‘flat’ position without restoration of the natural lordosis at the base of the lumbar spine [56]. Thus, after a rapid popularization period, the AxLIF is now rarely performed [55].

Other new emerging technologies include augmented reality, which displays three-dimensional spinal anatomy in real time over the patient’s anatomy. This supplies surgeons with additional information on depth, trajectory, and angulation in real-time and has now been applied to thoracolumbar pedicle screw placement, cervical screw placement, and interbody fusion [57,58,59]. The introduction of virtual reality in spine surgery allows spine surgeons to better plan surgical operations and can also assist with patient education [60]. As these technologies are in their infancy, further refinements and clinical data are needed prior to widespread adoption.

Another challenge in the adoption of minimally invasive spine surgery hinges on appropriate patient selection and appropriate surgical planning. While open approaches can be applied to all spinal pathologies, deciding which pathologies meet criteria for llowing minimally invasive intervention, especially within the confines of the operator’s skillset, is a much more nuanced process. Historically, this has relied heavily on surgeon’s experience, gestalt, prior failures, and prior successes. For novice minimally invasive surgeons, the zone of proximal development leans heavily towards “routine” pathology whereas, for the master surgeon, the threshold to convert to open surgery may be much higher. Consequently, there is much ambiguity, especially in the literature, regarding the “appropriate” candidate for minimally invasive surgery. This problem has not gone unrecognized, especially as it pertains to spinal deformity. In a subspecialty historically dominated by traditional open surgery, it was not until recently that surgeons formally published on minimally invasive deformity correction. In 2014, Mummaneni et al. of the Minimally Invasive Surgery Section of the International Spine Study Group published a landmark paper with the goal of providing a reproducible framework for decision-making and technique selection for patients with spinal deformity. This minimally invasive spinal deformity surgery (MISDEF) algorithm helped to guide surgeons on operative decision-making based on a variety of factors, including coronal cobb, fixed versus flexible deformity, sagittal balance, pelvic tilt, and lateral spondylolisthesis [61]. While this was based solely on an expert-panel of deformity surgeons representing both “open” and “minimally invasive” dichotomies, it was the first paper of its kind to provide a standardized framework and rationale behind operative decision-making. In 2019, updated minimally invasive techniques, such as anterior column release (ACR), mini-open pedicle subtraction osteotomy, and expandable cage technology, were incorporated into the algorithm, resulting in MISDEF 2 [62]. There is now even an entire textbook dedicated to decision-making in minimally invasive spine surgery [63]. 

## 4. Shifting the Paradigm

In the early days of minimally invasive spine surgery, a large barrier to adoption was the lack of powerful data demonstrating its clinical utility. Many surgeons were reluctant to endure a learning curve and purchase additional equipment for surgeries that had yet to be “proven”. Additionally, surgeons desired data proving superiority rather than simply equivalency. Fortunately, the ensuing decades demonstrably showed that minimally invasive surgery could reduce morbidity, pain, hospital costs, and even complications [32,64]. In a cost analysis study, Lucio et al. demonstrated an average cost savings of $2825.37 (10%) when minimally invasive surgery techniques were used instead of open techniques in the treatment of lumbar stenosis with instability [65]. For spinal deformity, minimally invasive approaches also resulted in significantly shorter stay in the intensive care unit as well as fewer blood transfusions and, while not statistically significant, patients undergoing minimally invasive deformity correction also had shorter total hospital stays by nearly 2 days [66].

Surgeons wishing to adopt minimally invasive spine surgery could thus provide the hospital with tangible evidence of clinical and financial utility. Coupled with hospitals’ desires to reduce costs, this facilitated the capital expenditure and material acquisition necessary to perform minimally invasive surgery. Secondarily, this motivated hospitals to seek surgeons with minimally invasive training. Furthermore, patients themselves became increasingly interested in minimally invasive alternatives, thus driving a more competitive market for minimally invasive spine surgeons [67]. These factors have contributed to the continuous growth of minimally invasive spine surgery, with additional projected growth through at least 2031 [68,69].

In the modern spine surgery landscape, there are still significant barriers to adopting minimally invasive spine techniques. This is particularly true for surgeons who did not initially train in programs focusing on proficiency in minimally invasive approaches. Residents and fellows who selectively train on programs with a heavy emphasis on minimally invasive techniques can receive supervised and gradual training in minimally invasive techniques, patient selection, complication avoidance and management. However, surgeons who did not have this luxury often find themselves alienated without a reliable pathway to adoption. While there is no perfect formula, the authors believe there are certain key tenets to adoption that must remain at the forefront. This includes surgeon education, which includes clinical data and mentorship, as well as technical training, which includes cadaver sessions, surgical simulators, and proctored cases. Additionally, industry partners can be an important ally, as many minimally invasive techniques are heavily dependent on vendors to provide the necessary equipment required for many newer surgical approaches. Lastly, there must be deliberate investment in funding and propagating fellowship programs that have emphasis on minimally invasive techniques. Without these tenets, it is difficult to visualize a future where minimally invasive spine surgery continues to grow and thrive.

Adoption of minimally invasive surgery is also regionally dependent. In a survey study from Lewandrowski et al., minimally invasive spine surgery was considered mainstream in up to 72.8% of practices in Asia and 70.2% in South America, but this number fell to 50% in Africa and the Middle East. While there were fewer respondents from the latter region, the differences in rates still reached statistical significance. Additionally, their data suggest that surgeons who employ minimally invasive techniques will do so for the majority of their surgeries, indicating a spine surgery practice pattern that emphasizes minimally-invasive-first principles [70].

In addition to regional differences, there has also been a steady migration of MISS towards outpatient surgical centers. With the adoption of Enhanced Recovery After Surgery principles, patients undergoing minimally invasive spine procedures are often able to be discharged less than 24 h after their procedure [71]. From an economic perspective, performing surgery at an outpatient center not only reduces hospital costs but can also increase operative efficiency whilst providing highly subspecialized nursing care. These centers are already immensely successful in orthopedic surgery and plastic surgery. The advancement of minimally invasive spine surgery also offers new potential in this realm [72].

## 5. Educating the Future

Surgeons have always recognized the importance of education and training. In her 2017 Presidential Address at the American College of Surgeons, Barbara Bass remarked, “Over this last decade or two, you often worked under the watchful eye of trusted mentors who brought you along all those years. Keep in touch with them…and pass on what they gave to you.” [73] For minimally invasive spine surgery, education and mentorship are equally important. That same year, the AOSpine Education Commission (AOSEC) conducted a global task force meeting with a specific focus on the development of a minimally invasive spine curriculum. Their panel of surgeon experts published the first-ever list of competencies and outlined the foundational minimally invasive procedures and general skills required to safely perform minimally invasive spine surgery (Table 2 and Table 3 [74]). Some of these were perceived as too specific, while others were deemed too basic. Nonetheless, this is still one of the first official forays into formalizing education and competency within minimally invasive spine surgery and serves as a set of guidelines for course and curriculum development within industry and fellowship programs.

As competencies and skillsets were published, data emerged on the learning curve for various surgeries. While the learning curve varied highly based on complexity, novelty, and surgeon’s prior experience, it became clear that each new technology was intricately associated with a learning process that was inherently defined by longer operative times and a higher rate of complications [75]. This led to the advancement and popularization of surgical simulators. These “ex vivo” training modalities allowed novice surgeons to hone their techniques, gain competency in procedures, and even to manage complications [76]. Today, surgical simulators are rapidly gaining popularity as they allow for “risk-less” training and familiarization with instrumentation, techniques, complication avoidance and complication management.

Additionally, teaching hospitals are now employing milestone-based measures for skill adoption. Whereas surgical education was once purely an apprenticeship model with large variances in surgical competency, nowadays, programs can utilize concrete measures to define skill growth and independence. One such example of this application can be seen at Duke University Health System, whose neurosurgery department utilizes the Surgical Autonomy Project (SAP) to provide bi-directional feedback and skill grades for commonly performed neurosurgical procedures [77]. Within the sphere of minimally invasive surgery, a tubular microdiscectomy would be segmented into four parts (i.e., Part 1: tube placement and docking, Part 2: laminectomy, Part 3: removal of ligamentum and dural exposure, Part 4: discectomy), which are each then further segmented into varying grades of independence (i.e., Zone 1: observation, Zone 2: performance with direct supervision, Zone 3: performance with minimal supervision, Zone 4: operator independence). Collection of data across time has allowed faculty members to better gauge the skill level of their trainees, and helps trainees understand their areas of strength and weakness [78].

Lastly, no review on minimally invasive surgery can be complete without discussion of robotics and 3-dimensional CT-based navigation. At the turn of the new millenia, intraoperative CT and spinal stereotaxy became widely available. These technologies have been able to reduce the learning curve for instrumentation, improve pedicle screw accuracy, and facilitate deformity correction [79,80,81]. With each iterative improvement in both software and hardware, robotics and navigation are seeing new indications. Such novelties include the usage of these technologies for interbody placement, osteotomy planning, and tumor resection [82]. While future surgeons will still need mastery of free-hand or fluoroscopy-based instrumentation, it is imperative that the new generation also train with these new technologies. In the future, it is certainly possible that navigation and robotic surgery become the standard-of-care.

## 6. Conclusions

**Figure 4:**
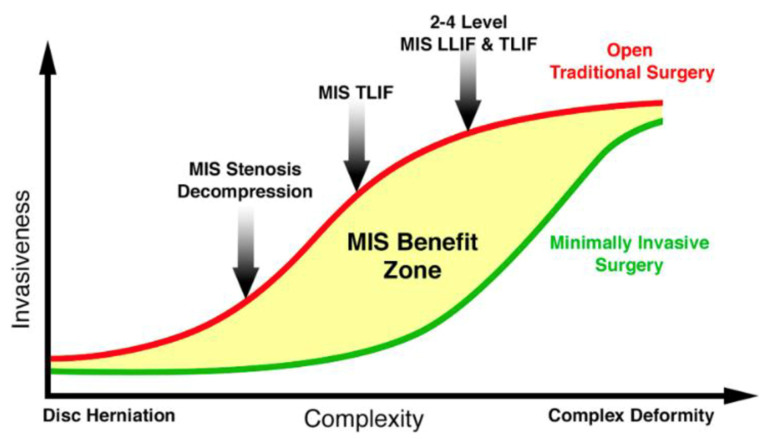
From Hartl 2020 [83]. The “benefit zone” for MISS includes procedures in the mid-zone of complexity, for example, MIS stenosis decompression, MIS–TLIF, or 2- to 4-level MIS LLIF and TLIF. Posted with permission under the terms of the Creative Commons Attribution-NonCommercial-NoDerivs 4.0 License.

In April 2020, Roger Hartl published a landmark editorial outlining a platform for the advancement of minimally invasive spine surgery [83]. He coined his framework as “The 6 T’s of Minimally Invasive Spine Surgery”, which discussed the importance of combining a well-trained surgeon (Training, Talent) with the optimal pathology (Target) with the best surgical approach (Technique, Testing) and enabling technology (Technology). These cases fell into what Hartl coined as the “MIS Benefit Zone” (Figure 4). As we continue into the second quarter of the 21st century, it will become increasingly important for surgeons to remain introspective and self-aware. In this age of exponential growth, surgeons must remain vigilant to effectively delineate between worthwhile advancements from the spectacle of misguided technology. As new techniques, technology, and surgeon training aim to expand the MIS Benefit Zone, it is still important to recognize that there are many pathologies where patients may experience maximum benefit from traditional open surgery. Whether this be gained through reductions in operative time, more substantial deformity correction, or more thorough tumor resection, for example, varies obviously from case-to-case, but applying minimally invasive surgery as a blanket approach may result in patients who receive suboptimal care. As surgeons, we must recognize that the ultimate goal of any intervention is still to provide the best care and outcome that is possible for each of our patients.

## Figures and Tables

**Table 1:** Technical milestones across the years.

Time	Advancement	Advantages	Disadvantages
1968	Wiltse Approach	Intramuscular dissection, pioneering, tissue-preserving	Poor cosmesis, limited exposure
1980s	Trans–Kambin Approach	Percutaneous discectomy, ultra-minimally invasive	Possible injury to dorsal root ganglion, reliant on fluoroscopy
1990s–early 2000s	Tubular approaches	Allows direct decompression, minimal tissue disruption, easily adaptible	Unfamiliar visualization, limited exposure
Early 2000s	CT-navigation	Accurate, widely available, reduces occupational radiation	Costly, increased radiation for patient, space-occupying
2006	Lateral-access	Indirect decompression, minimal blood loss, deformity correction	Femoral nerve palsy, possible peritoneal injury, psoas hematoma
Mid–2000s	Robotics	Reproducible, minimizes learning curve, indications expanding	Expensive, limited indications, potentially increases operating room time
2000s	Endoscopy	Ultra-minimally invasive, allows for awake surgery	Steep learning curve, equipment and capital investment, limited instrumentation
2010s	Augmented Reality	Familiar anatomic visualization, integrates with existing instrumentation	Accuracy, increased operative time, bulky headset

**Table 2:** AOSpine Competencies for Minimally Invasive Spine Surgery.

AOSpine Competencies for Minimally Invasive Spine Surgery
1. Diagnose the patient problem by correlating the clinical finding with imaging and workup
2. Recognize appropriate indications based on your skill set, case experience, and outcomes
3. Select the appropriate MISS procedure for the pathology and indication, and recognize when MISS is not the appropriate option
4. Correctly set up the technology, operating room, and the team of the procedure
5. Perform microscopic minimally invasive procedures: posterior cervical foraminotomy, interlaminar lumbar discectomy, lumbar extraforaminal discectomy, and unilateral laminotomy for bilateral decompression
6. Perform endoscopic procedures: interlaminar lumbar discectomy, transforaminal lumbar foraminotomy and discectomy, and unilateral laminotomy for bilateral decompression
7. Perform the fusion MISS procedures (percutaneous screws and rod placement, (transforaminal lumbar interbody fusion, TLIF) and lateral lumbar interbody fusion (LLIF)) and apply strategies to optimize arthrodesis
8. Manage complications and apply a backup plan
9. Use MISS techniques for revision surgery

**Table 3:** AOSpine Foundational Procedures and Skills for Minimally Invasive Spine Surgery.

AOSpine Foundational Procedures and Skills for Minimally Invasive Spine Surgery
Procedures
1. Interlaminar microscopic tubular lumbar discectomy (IMTLD)
2. Posterior microscopic tubular cervical foraminotomy (PMTCF)
3. Extraforaminal microscopic tubular lumbar discectomy (EMTLD)
4. Interlaminar endoscopic lumbar discectomy (IELD)
5. Transforaminal endoscopic lumbar foraminotomy and discectomy (TELF/TELD)
6. Lumbar endoscopic unilateral laminotomy for bilateral decompression (Endoscopic “over the top” decompression or endoscopic LE–ULBD)
7. Microscopic tubular unilateral laminotomy for bilateral decompression (“over the top” decomp, MT–ULBD)
8. Percutaneous screw and rod placement
9. MIS transforaminal lumbar interbody fusion (TLIF)
General Skills
1. Using a microscope
2. Using an endoscope
3. Using a burr with an endoscope
4. Using a drill for minimally invasive spine surgery (MISS)
5. Using 2D and 3D navigation and assistive technologies
6. Managing a dural tear
7. Bleeding control
8. Radiation reduction
9. Placing a tubular retractor (or retractor)

## Data Availability

No primary data were involved in this paper.
